# Effectiveness of an Interactive School-Based Oral Health Educational Program on Periodontal Status Among Palestinian Adolescents: An Intervention Study

**DOI:** 10.3390/children12101302

**Published:** 2025-09-26

**Authors:** Sura Al-Hassan, Mazen Kazlak, Elham Kateeb

**Affiliations:** 1 Faculty of Public Health, Al-Quds University, Jerusalem 51000, Palestine; 2 Faculty of Medicine and Health Sciences, An-Najah National University, Nablus 00970, Palestine; mazen.kazlak@najah.edu; 3 Oral Health Research and Promotion Unit, Faculty of Dentistry, Al-Quds University, Jerusalem 51000, Palestine

**Keywords:** effectiveness, oral health education, school students, oral hygiene practices, adolescents, Nablus

## Abstract

**Background & Objectives**: Periodontal disease is a common but preventable condition characterised by chronic inflammation of the periodontium caused by microbial infection. School-based oral health education can promote healthy behaviours and enhance periodontal health. This study was to assess the effects of an interactive oral educational program on periodontal status, oral hygiene, and related behaviours among 9th-grade students in Nablus City. **Method**: A pre-test/post-test experimental design was conducted from 2023 to 2024 in governmental and private schools. A stratified random sampling procedure selected 536 students for the intervention group and 410 for the control group. Baseline and two-month follow-up data were collected via a self-administered questionnaire and clinical examinations using the Community Periodontal Index for Treatment Needs (CPITN) and the Simplified Oral Hygiene Index (S-OHI). Statistical analysis (chi-square test; paired and independent *t*-tests) was performed with significance set at *p* < 0.05. **Results**: At follow-up, the intervention group showed significant reductions in CPITN (from 10.99 ± 2.77 to 10.00 ± 2.64; *p* < 0.001) and S-OHI (from 12.90 ± 3.10 to 10.89 ± 2.78; *p* < 0.001). Significant improvements were also observed in oral hygiene practices, dietary habits, and smoking behaviour scores (all *p* < 0.001). No significant changes occurred in the control group. **Conclusions**: The interactive, school-based oral health education program effectively improved periodontal health, oral hygiene status, and related behaviours among adolescents.

## 1. Introduction

Periodontal diseases (PD) are inflammatory conditions that affect the tissues that support the teeth within their sockets [1,2]. These diseases are primarily caused by bacterial plaque accumulation [1,2,3]. PD ranges in severity from gingivitis to periodontitis [4]. Gingivitis, the mildest form of PD, is characterised by inflammation of the gum tissue without affecting the underlying bone and is typically reversible. However, when left untreated, gingivitis can progress to periodontitis, a more severe condition that leads to the destruction of the supporting bone and connective tissue. Periodontitis is characterised by the formation of pockets around the teeth that can lead to tooth loss [5]. The pathogenesis of PD is multifactorial, involving a complex interplay of immunological, genetic, and environmental factors [6]. It arises from an imbalance between the host and the resident microbiome, leading to microbiome dysbiosis. This dysbiosis triggers an upregulated inflammatory response in the host, which contributes to the destruction of the periodontal extracellular matrix [7,8].

Globally, PD affects approximately 20% to 50% of the global population [9]. Gingivitis is particularly prevalent among older children and adolescents, with only 21% of adolescents showing no signs of PD. Approximately 18.8% exhibit bleeding on probing, while 50.3% demonstrate the presence of calculus [10].

In developing nations, the prevalence of calculus and bleeding on probing among adolescents is notably higher. The proportion of adolescents with gingivitis ranges from 35% to 70% in these regions, compared to 4% to 34% in developed nations [11]. The prevalence of gingivitis varies significantly among adolescents across countries. It was most common in Norway (66%), followed by Iran (30%) and Belarus (15%) [10]. While in China, the prevalence of gingivitis among 12–15-year-old children was 29.6%, with 22.6% having localised gingivitis and 7.0% having generalised gingivitis [12]. In Uttarakhand, India, 20.0% of adolescents had gingival bleeding, and 5.4% had severe gingivitis [13]. In southern Jordan, oral hygiene among students in Tafelah schools is generally considered fair, with a notable prevalence of mild to moderate gingivitis. The findings showed that only 29.8% of students have healthy gums, while 38.5% display mild gingivitis, 31.4% have moderate gingivitis, and 0.3% experience severe gingivitis [14]. In Gaza, high school students showed a notably high average gingival index (GI) of 1.5 ± 0.80. Among the recorded cases of gingivitis, 28.5% were classified as mild, 44.5% as moderate, and 27% as severe [15]. Similarly, in the north of the West Bank, only 13% of 9th-grade students had healthy gums, while 44% exhibited gingival bleeding and 42% had calculus [16]. However, when PD is detected in its reversible stage, it can be reversed to a healthy periodontal condition through the control of modifiable risk factors, including poor oral hygiene and smoking [1,8,17].

Oral health education is a cost-effective strategy that can mitigate the negative impact of periodontal risk factors by promoting appropriate dietary and oral hygiene practices, ultimately reducing the incidence of PD [18,19]. Squarely, educational programs can affect various age groups, benefiting both adolescents and adults [20,21]. In adolescents, such programs primarily focus on promoting proper oral hygiene practices, emphasising the importance of a balanced diet, outlining ways to prevent dental problems, and highlighting the harmful effects of smoking [22,23]. Indeed, education designed to improve the periodontal health of adolescents [24] typically provides specific information about gum structure, the adverse effects of unhealthy habits such as smoking, and the connection between gum diseases and systemic conditions [11,25,26]. School-based educational programs remain an effective venue for oral education [27] by enhancing students’ access to dental screenings, particularly those from socioeconomically disadvantaged backgrounds [28]. It offers a unique opportunity to develop the personal skills necessary for a healthy lifestyle [24,29]. This school-based education often includes classroom presentations and dental screenings [30]. While interactive-based school education, which includes engaging approaches like games and innovative techniques, has produced better outcomes in terms of plaque and gingival index scores, as well as oral hygiene knowledge and practices, when compared to traditional oral health promotion methods [31,32].

Paying attention to PD across all stages of life is essential. However, adolescents demand more focus due to their hormonal changes [33]. Additionally, adolescents are at greater risk of developing poor dental and dietary habits compared to younger children, making them more susceptible to PD. In turn, adolescents need specific oral preventive strategies [34,35]. Notably, age 15 is considered pivotal, as it represents an index age for international comparisons and monitoring PD trends [36].

Thus, this study aimed to assess the effectiveness of a two-month interactive educational program in periodontal status among 15-year-old schoolchildren in Nablus, one of the most urbanised cities in the northern West Bank. This study utilised commonly employed PD indices, including the Community Periodontal Index for Treatment Needs (CPITN) and the Simplified Oral Hygiene Index (S-OHI). Additionally, this study assessed the effect of the interactive educational program on oral hygiene status and practices, dietary habits, and smoking behaviours among adolescent participants.

To the best of the researcher’s knowledge, limited attention has been given to PD in Palestine, and there are no published studies evaluating the effectiveness of school-based oral health education programs in the region. This study builds on previous work [16] that explored the prevalence of PD among adolescents and identified the determinants contributing to its high burden. This study hypothesised that there would be differences in CPITN, SOHI, oral hygiene practices, dietary habits, and smoking behaviours between the intervention and control groups after two months of implementing an interactive oral health educational program.

## 2. Materials and Methods

### 2.1. Study Design, Population, and Setting

A pre-test/post-test experimental design was utilised in this study. This design was chosen to achieve internal validity for the study [37,38,39]. An interactive educational approach was adopted to teach oral health self-care. This approach actively engaged participants through two-way communication, practical demonstrations, and hands-on activities. During the sessions, the students were encouraged to ask questions, participate in discussions, and practice real-time skills. This dynamic approach was chosen to enhance participants’ understanding of proper oral health practices and foster positive behaviour change [40].

### 2.2. Sample Techniques and Process

This study employed a multistage sampling technique to ensure a representative sample of students from Nablus City schools. A stratified simple random sampling method was used to select both schools and students. Schools were stratified based on their geographical location (East, Central, West), and a proportional number were randomly selected from each stratum (Table 1).

The eligibility criteria for schools to participate in this study were the presence of 9th-grade classes, feasibility for participation, and proper consent procedures for both students and parents. Based on the total number of eligible schools (East = 27, Central = 25, West = 13), 15 schools were randomly selected using Epi Info: 6 from the East, 6 from the Central, and 3 from the West. Among them, 10 were governmental and 5 were private schools. These 15 schools were then randomly allocated into two groups: one for the intervention and one for the control. Randomisation was performed at the school level to reduce contamination, while ensuring geographic and type-based balance. This rigorous sampling and allocation approach reduced potential selection bias and confounding factors that could affect the relationship between the intervention and outcomes [39].

According to the 2024 records of the Nablus Educational Directorate, there were 2832 ninth-grade students in governmental schools (1360 males and 1472 females) and 737 students in private schools (434 males and 303 females). Following methodological guidance on a priori sample-size planning with G*Power version 3.1.9.7 [41,42]. A *t*-test as the primary statistical method, an assumed medium effect size (dz = 0.5), a significance level (α) of 0.05, and a statistical power of 90% (1 − β = 0.90) were adopted. This ensured the study was adequately powered to detect meaningful differences while minimising the risks of Type I and Type II errors.

**Figure 1:**
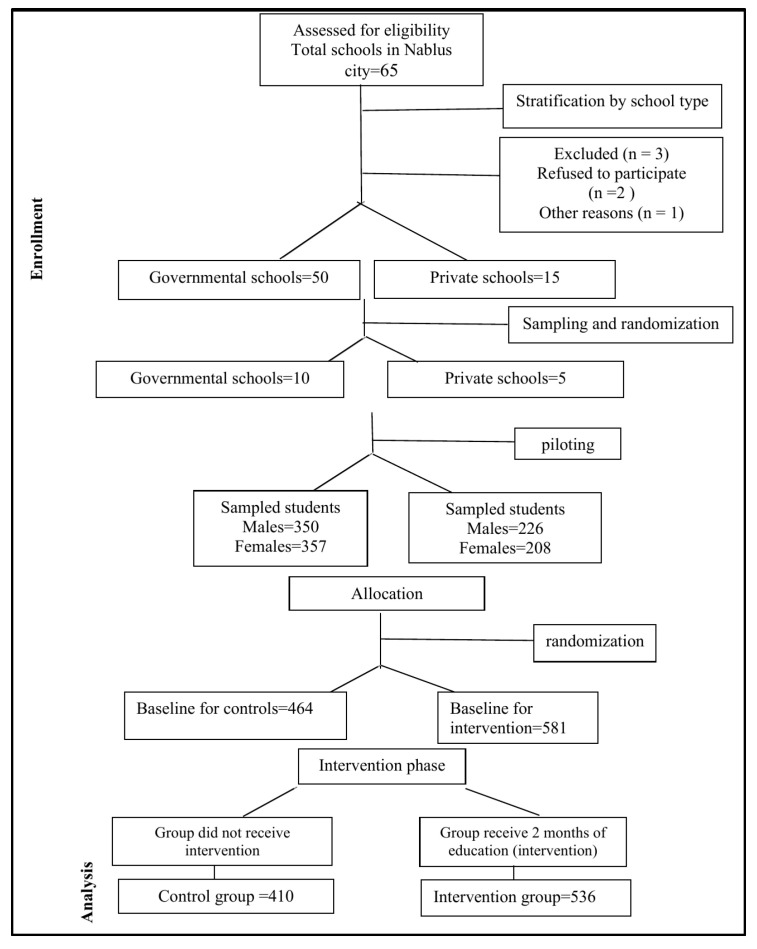
CONSORT flow diagram showing the flow of schools and students selected through each stage of the trial.

Based on these parameters and the logistical feasibility of data collection, the final sample included 536 students from the intervention group and 410 students from the control group. This distribution reflects the actual number of students available and willing to participate in each group, with a slight intentional preference to include more students in the intervention group to enhance the study’s ability to detect effects of the educational program. (See Figure 1: CONSORT flowchart of student recruitment and screening).

### 2.3. Pilot Study

Following established guidelines for conducting a complete study [43], a pilot survey was conducted with 10% of the predetermined sample (100 students). The pilot’s study primarily focused on assessing the feasibility of the research process and the students’ comprehension of the questionnaire items. Additionally, conducting a descriptive analysis without testing any hypotheses related to clinical outcomes [44]. In the pilot stage, the questionnaire’s face and content validity were evaluated by three experts in the field. Following this assessment, the questionnaire was pre-tested with participants to confirm its relevance and comprehensibility (face validity) [45]. Additionally, this step ensured that the questionnaire thoroughly addressed all aspects of the study variables (content validity). Based on the feedback received from the pilot study, the final version of the questionnaire was refined [46]. An Exploratory Factor Analysis (EFA) was performed using Principal Component Analysis to evaluate the instrument’s construct validity. The suitability of the data for factor analysis was assessed using the Kaiser-Meyer-Olkin (KMO) measure of sampling adequacy and Bartlett’s Test of Sphericity. The KMO value was 0.70, indicating satisfactory sampling adequacy, and Bartlett’s test was significant (*p* < 0.001), which supports the factorability of the correlation matrix. Factors were extracted based on eigenvalues greater than 1 and by inspecting the scree plot. The analysis identified a 4-factor solution that explained 71.30% of the total variance. Items were retained if they had factor loadings of 0.50 or higher and had theoretical relevance. The data collected during the pilot study were not included in the final analysis.

### 2.4. Clinical Screening

In this study, the Community Periodontal Index for Treatment Needs (CPITN) was used to assess the prevalence of periodontal disease (PD) among 9th-grade students before and after the intervention. CPITN is widely recognised as a reliable tool for evaluating PD prevalence, and its results are often used to dictate health policy and develop strategic plans for PD control programs [10,47,48]. The operational characteristics of CPITN, including its comparison with standard examinations and diagnostic criteria, have been evaluated in previous studies. Findings indicated that CPITN has a sensitivity of 58%, a specificity of 80%, and positive and negative predictive values of 87% and 46.3%, respectively [49]. Additionally, the validity of CPITN has been confirmed in the early detection of gingivitis and periodontitis [50].

Aligning with World Health Organisation (WHO) guidelines [51], this study adopted the CPITN scores for adolescent screening, which include score 0: healthy gingiva, score 1: gingival bleeding observed after gentle probing, and score 2: presence of supra or sub-gingival calculus. The index teeth that represent the participant’s mouth sextants were: 16, 11, 26, 36, 31, and 46.

The Oral Hygiene Index-Simplified (OHI-S), developed by Greene and Vermillion [52], was used to determine the oral hygiene status of the subjects. The index comprises two components: the Debris Index–Simplified (DI-S), representing the soft plaque index, and the Calculus Index–Simplified (CI-S), representing the calcified plaque index. Debris Index–Simplified (DI-S)—Soft Plaque: Score 0: No soft debris or extrinsic stain, 1: Soft debris covering not more than one-third of the tooth surface, Score 2: Soft debris covering more than one-third but not more than two-thirds of the tooth surface, Score 3: Soft debris covering more than two-thirds of the tooth surface. While the Calculus Index–Simplified (CI-S)—Calcified Plaque have the following scores: Score 0: No calculus present, Score 1: Supragingival calculus covering not more than one-third of the tooth surface, Score 2: Supragingival calculus covering more than one-third but not more than two-thirds of the tooth surface, or isolated flecks of subgingival calculus around the cervical portion of the tooth, Score 3: Supragingival calculus covering more than two-thirds of the tooth surface, or a continuous heavy band of subgingival calculus around the cervical portion of the tooth. Six index teeth (16, 11, 26, 36, 31, and 46) were examined on specific surfaces (buccal for upper molars and lower incisors, lingual for lower molars, and labial for upper incisors). For each participant, DI-S and CI-S scores were calculated by summing the scores for the examined surfaces and dividing by the number of surfaces examined. The OHI-S score was obtained by adding the DI-S and CI-S scores, yielding a range from 0 to 6, with higher scores indicating poorer oral hygiene.

In the pilot phase of the study, the Interclass Correlation Coefficient (ICC) was calculated to assess the reliability of the clinical measurements. A total of 20 students were examined five times by five data collectors, resulting in ICC values of 0.83 for CPITN and 0.98 for OHI-S, indicating high reliability.

### 2.5. Instrument Development

The questionnaire used in this study was adapted from existing literature [36,53,54], with modifications to evaluate students’ oral hygiene practices, dietary habits, and smoking behaviours before and after education. Initially, the questionnaire was developed in English and later translated into Arabic. To ensure accuracy, the translation was validated through a back-translation process from Arabic to English before being administered as a pre-test.

The questionnaire was divided into five sections: student background, which included questions about the type of school (governmental or private), family background, which covered family size and economic status, parents’ educational level, and parents’ occupational status. While the oral hygiene practices section addressed the frequency and technique of tooth brushing, the dietary habits section focused on the frequency of breakfast consumption per week and the daily intake of nutritious versus non-nutritious foods. The smoking behaviours section assessed the frequency of cigarette and water pipe smoking and the number of cigarettes usually consumed by the student per week. The questionnaire’s internal consistency was evaluated using Cronbach’s alpha, yielding a reliability coefficient of 0.967 for the 47 items tested. The questionnaire was administered through direct interviews with individual students. The response rate was 95%.

### 2.6. Data Collection Processes

The Al-Quds Committee for Ethical Considerations approved this study (REF.13/24). Subsequently, the study was registered at ClinicalTrials.gov (Identifier: NCT07055932). Then, consent forms were distributed to the parents of students, and their approval was sought before data collection began. To ensure the data collection process was standardised and calibrated, the principal researcher trained five dentists to conduct interviews and dental examinations. Training sessions for the data collectors were held every 15 days throughout the research period to maintain consistency and accuracy in data collection. All the participating students received a detailed explanation of the study’s main objectives at the start of the baseline phase. Baseline data were collected in March 2024. After two months of education, follow-up data were collected in the same schools using the same dental examination measures and questionnaires used during the baseline phase (See Supplementary Figure S1).

### 2.7. Intervention Description

The oral education program in this study focused on periodontal tissues and their conditions. It aims to equip participants with the knowledge and skills to manage and prevent periodontal problems. The program was adapted from previous studies, with modifications made to suit the specific objectives of this research [55,56]. The educational content was based on the standard guidelines set by the WHO [36]. The control group received one oral educational session, while the intervention group participated in six sessions. The educational sessions were held every 10 days for two months. The education sessions, which lasted between 30 and 45 min, employed an interactive approach, incorporating multiple audiovisual aids such as dental forms, models, charts, posters, and plaster models (Table 2). These materials were designed to engage participants actively and foster a deeper understanding of proper oral health practices.

### 2.8. Blinding

To minimise bias, blinding is commonly applied in clinical and field research through strategies such as participant blinding, outcome assessor blinding, and blinding of data collectors, who may administer diagnostic tests, conduct interviews, or record outcomes [57,58]. In the present study, two levels of blinding were implemented. First, participant blinding was achieved, as the participant students in the intervention group were not aware that there was another group that received only a single educational session, and students in the control group were equally unaware of the intervention structure in other schools. In other words, participants had no information about the existence of different study groups or the number or nature of sessions delivered elsewhere, and this aligns with participants’ binding level in educational interventions [59,60,61]. Second, blinding of data collectors was maintained as assessors were unaware of the participants’ group assignments. This minimised detection and ascertainment bias following random allocation [62]. However, blinding the intervention provider (the researcher delivering the educational content) was not feasible due to the nature of the intervention. Based on standard definitions of blinding in intervention research [62,63,64], this study qualifies as a double-blind design, as both participants and outcome assessors were blinded to the group allocation.

### 2.9. Outcomes

The original responses were re-coded into continuous variables. The primary outcome measures were the changes in the mean scores of CPITN, S-OHI, oral hygiene, dietary habits, and smoking behaviours immediately after the two-month intervention in the interventional group.

### 2.10. Data Management and Statistical Analysis

During the data collection phase, records were reviewed daily for completeness and consistency. Data was analysed using IBM SPSS Statistics version 25.0. Descriptive statistics were utilised to summarise the baseline socio-demographic characteristics of each group, ensuring their comparability at the start of the study.

Given the pre-post intervention design of this study, which involved comparing the means of the CPITN and S-OHI scores both within and between the two independent groups, appropriate statistical methods were selected to address the research objectives, which were to evaluate the effect of education on periodontal and oral hygiene status, as well as on other oral health-related behaviours. Paired sample *t*-test was used to assess within-group changes over time (pre- vs. post-intervention), as this method is suitable for repeated measurements on the same subjects [65,66]. An independent sample *t*-test was employed to evaluate differences between the intervention and control groups at each time point, since the groups were mutually exclusive and independently randomised [65]. To ensure the appropriateness of these parametric tests, the Kolmogorov–Smirnov test was conducted to examine the normality of continuous variables. The results confirmed that the distributions were suitable for the *t*-test application. Moreover, each group had a relatively large sample size (*n* > 30), which supports the robustness of *t*-tests [67]. Results were reported as mean ± standard deviation (Mean ± SD), mean differences (MD), t-values, *p*-values, and 95% confidence intervals (CI).

To evaluate the localised impact of the intervention [51], independent sample *t*-tests were used to compare the mean CPITN and S-OHI scores between groups across participants’ mouth sextants. This analysis facilitated the evaluation of regional differences in periodontal and oral hygiene status, enabling the determination of the statistical significance of intergroup differences in specific mouth regions.

On the other hand, questionnaire responses related to oral hygiene, dietary, and smoking variables were re-coded into scale variables to facilitate the computation of mean scores before and after the intervention [68,69,70,71]. Correct answers reflecting preventive behaviours, in line with American Dental Association guidelines [72], were assigned the highest scores. At the same time, incorrect responses received the lowest scores, for example: “Do you clean your teeth?”: Yes = 1, No = 0, “How often do you clean your teeth?”: Once/day = 1, Twice/day = 2, Three times/day = 3, “How long does it take to clean your teeth?”: One minute = 1, Two minutes = 2, More than two minutes = 3, Don’t know = 0, “Brushing technique”: Scrubbing = 1, Bass = 2, Modified Bass = 3, “Toothbrush type”: Hard bristle = 1, Soft bristle = 2, Medium bristle = 3, “Toothbrush replacement frequency”: Every three months = 1, Two months = 2, Monthly = 3 (See Appendix A).

The overall mean differences in oral hygiene practices, dietary habits, and smoking behaviour scores were compared between groups using independent sample *t*-tests, with sub-analyses conducted for each behavioural domain separately.

To isolate the specific effect of the intervention beyond natural variation or time-related changes, the net effect (difference-in-differences) approach was applied. The approach is widely used in public health intervention studies and is considered a valid method for evaluating program effectiveness in school-based designs [73]. This method is well-suited for pre-post-controlled designs. In this study, the net effect was calculated by subtracting the pre-post difference in the control group from the pre-post difference in the intervention group for the means of CPITN, S-OHI, oral hygiene, dietary, and smoking scores. This allowed us to estimate the impact of the oral health education program while controlling background trends [74]. Statistical significance was set at *p* < 0.05.

## 3. Results

At the baseline phase, the sample comprised 53.3% males in the control group and 47.7% in the interventional group. In comparison, females accounted for 46.7% of the control group and 52.3% of the interventional group. Most participants in both groups attended governmental schools, with 74.2% in the control group and 77.5% in the interventional group. The distribution of socioeconomic status among participants (categorised as excellent/very good, good, moderate, and low) indicated that most individuals in both groups reported a moderate socioeconomic status, which comprised 54.1%.

The control and intervention groups were comparable across almost all socio-demographic factors: sex (*p* = 0.089), type of school (*p* = 0.247), socioeconomic status (*p* = 0.140), number of siblings (*p* = 0.293), father’s employment status (*p* = 0.156), mother’s employment status (*p* = 0.175) and father’s education level (*p* = 0.978), all showed no statistically significant differences (*p* > 0.05). The only exception was the mother’s education level, which differed significantly between the two groups (*p* < 0.001), indicating that the distribution of maternal education was not equivalent at baseline (Table 3).

No significant differences between the control and intervention groups were observed in the mean of CPITN and S-OHI scores at baseline (*p* = 0.407 and *p* = 0.276, respectively). After two months of education, the intervention group demonstrated a significantly lower mean CPITN score of 10.00 ± 2.639, compared to their baseline mean of 10.99 ± 2.766, and lower than the control group’s mean score after the intervention, which was 11.00 ± 2.582. These results were statistically significant, with a *p*-value of 0.000. Additionally, for the S-OHI mean scores, the intervention group had a lower mean score of 10.89 ± 2.779, compared to their baseline mean of 12.90 ± 3.103, and also lower than the control group’s mean score after the intervention, which was 12.53 ± 2.466 (Table 4).

The comparison of the means of CPITN scores across mouth sextants between the control and intervention groups shows that the intervention group exhibited a statistically significant reduction in several sextants compared to the control group. In the Lower Right Sextant, the control group had a mean score of 1.54 ± 0.617, while the intervention group recorded a mean score of 1.33 ± 0.602. The mean difference (MD) between the groups was −0.215.

In the Upper Middle Sextant, the control group had a mean score of 1.94 ± 0.594, whereas the intervention group presented a lower mean score of 1.61 ± 0.682, with an MD of −0.323.

For the Lower Middle Sextant, the control group had a mean score of 2.19 ± 0.756, while the intervention group showed a slightly lower mean score of 2.02 ± 0.814, resulting in a mean difference of −0.176.

In the Lower Left Sextant, the control group’s mean score was 1.50 ± 0.573, while the intervention group achieved a notably lower mean score of 1.28 ± 0.572. The mean difference was −0.221 (Table 5).

The findings indicate that all sextants showed lower mean scores of S-OHI in the intervention group compared to the control group. The most significant reduction was observed in the lower middle sextant, with a mean reduction of 0.379 (control group: 2.40 ± 0.751, intervention group: 2.03 ± 0.783), indicating the most significant decrease in the mean score. The second-highest reduction occurred in the lower right sextant, with a mean reduction of 0.336 (control: 1.79 ± 0.639, intervention: 1.45 ± 0.645). Conversely, the upper left sextant showed the least improvement, with a mean reduction of 0.154 (control: 2.25 ± 0.676, intervention: 2.10 ± 0.730) (Table 6).

At the baseline, there were no significant differences between the control and intervention groups regarding oral hygiene practices (P = 0.066), dietary habits (P = 0.563), and smoking behaviours (P = 0.071).

The intervention group demonstrated the most substantial improvement in combined practices—encompassing oral hygiene, dietary habits, and smoking behaviours—rising from a mean score of 54.75 ± 10.426 to 62.73 ± 9.834 (P = 0.000). In contrast, the control group showed only a minor improvement.

Oral hygiene practices in the intervention group significantly improved after two months, increasing from 13.60 ± 6.548 to 17.86 ± 5.931 (P = 0.000). Meanwhile, smoking behaviours in the control group exhibited minimal and non-significant changes, moving from 6.11 ± 3.429 to 6.40 ± 3.606 (P = 0.356) (Table 7).

The intervention group demonstrated significant improvements in various oral hygiene practices, particularly in brushing habits. Their mean brushing status score was 0.93 (±0.254), compared to the control group’s score of 0.83 (±0.375), indicating more consistent brushing habits (*p* = 0.000).

Additionally, the intervention group reported brushing their teeth more frequently, with a mean of 1.21 times (±0.563) per day, whereas the control group averaged 0.98 times (±0.593) per day (*p* = 0.000). They also brushed for a longer duration, with a mean of 1.14 min (±0.859) compared to the control group’s 0.70 min (±0.609) (*p* = 0.000) (Table 8).

A comparison of dietary habits between the control and intervention groups revealed that breakfast consumption habits were the only variable that showed a statistically significant difference. Participants in the intervention group reported higher breakfast consumption (Mean = 6.25 ± 2.44) compared to the control group (Mean = 5.83 ± 2.35), with a t-value of −2.703 and a *p*-value of 0.007 (Table 9).

The smoking status (yes/no) showed a significant reduction in the intervention group (Mean = 1.50, SD = 0.500) compared to the control group (Mean = 1.56, SD = 0.501), t = 2.043, *p* = 0.040.

The number of cigarettes smoked per day among participants in the intervention group (Mean = 8.04, SD = 3.560) was significantly lower than that in the control group (Mean = 11.05, SD = 4.218), revealing a statistically significant difference (t = 7.800, *p* = 0.000).

Regarding water pipe smoking frequency, the intervention group also reported a lower mean (Mean = 2.201, SD = 0.966) than the control group (Mean = 2.673, SD = 0.890). This difference was statistically significant (t = 7.778, *p* = 0.000). Although the difference in water pipe smoking status was minimal (Control Mean = 1.14 vs. Intervention Mean = 1.13), it was statistically significant (t = 0.740, *p* = 0.001), suggesting a slight but meaningful reduction in the proportion of water pipe smokers in the intervention group (Table 10).

CPITN showed a net reduction of −1.14, while the Simplified Oral Hygiene Index (S-OHI) improved by −1.85 in favour of the intervention group. Regarding health-related practices, the net effect was +4.10 for oral hygiene behaviours, +1.63 for dietary habits, and +0.44 for smoking behaviours. The cumulative improvement across all practice domains reached a net effect of +6.30 (Table 11).

## 4. Discussion

Studies on PD in Middle Eastern countries are limited [75,76], particularly in Palestine, where oral health issues, including PD, often do not receive attention as other chronic conditions [77]. However, early dental intervention can significantly mitigate the adverse effects of PD. For instance, with proper oral hygiene and periodontal healthcare, the annual incidence of tooth loss is only 0.1 teeth per patient, allowing many individuals to maintain their natural teeth for life [78,79]. Furthermore, the Arab region studies focus on the relationship between PD and its risk factors; thus, there is a gap in exploring the impact of educational programs on PD prevention [80,81]. For that, this study aimed to assess the effect of an interactive oral health educational program on periodontal health and oral hygiene status among 9th-grade students in Nablus City. Additionally, the study examined the program’s effect on students’ oral hygiene practices, dietary habits, and smoking behaviours two months after the education was implemented.

This study employed audio-visual aids and interactive practical demonstrations, such as coloured illustrations, models, puzzles, and lectures. This likely facilitated student engagement and enhanced understanding of proper oral health practices. This approach is consistent with the findings of Menaka et al. [82], which suggested that interactive methodologies are more effective than traditional methods in promoting changes in oral health behaviours. Additionally, this study was conducted in governmental and private schools—key formal settings where well-structured health programs can significantly improve students’ oral health [83].

This study adopted a robust design with a large sample size, stratified random sampling, and validated indices, including the CPITN and S-OHI. Furthermore, incorporating a double-blind approach and including a control group might enhance the reliability and validity of the findings.

At the baseline phase, the socio-demographic variables did not show significant differences between the control and intervention groups, except for the mothers’ education level. This observed difference could be attributed to the large sample size, which tends to increase the statistical power and sensitivity of tests. However, greater sensitivity enhances the ability to detect actual differences; it may also lead to statistically significant findings that lack clinical or practical relevance [84].

In the present study, minimal improvement was observed in the control group’s mean CPITN scores and some oral hygiene practices, such as brushing duration. This modest improvement may be attributed to the Hawthorne effect [85,86] or could result from the control group’s oral health baseline assessment, which may prompt them to modify their oral hygiene habits. Additionally, the presence of dental professionals in the participant students’ schools may have heightened their awareness of the significant role of improving their oral care. It is worth noting that some students may have sought additional information through social media or other sources.

After two months of education, the intervention group demonstrated statistically significant lower mean CPITN and S-OHI scores across all mouth sextants compared to both their baseline measures and the control group. These results confirm that the educational intervention was indeed capable of making a difference, as evidenced by the clear and positive shift in periodontal and oral hygiene indicators. This change in dental indices is likely attributed to the program’s interactive and consistent nature, which was delivered regularly over nearly two months. The interactive and participatory approach had enhanced students’ engagement, increased retention of take-home messages, and encouraged healthier oral health behaviours. The observed reductions in scores of both indices indicated improvements in periodontal and oral hygiene status, which was likely due to increased adoption of proper oral health care practices by the students (such as brushing and flossing techniques, frequency, and duration). These results show both within-group improvements (pre- vs. post-intervention in the intervention group) and between-group differences (intervention vs. control after the intervention), suggesting the program’s overall effectiveness and relative efficacy.

Compared to other studies, the intervention group in our study achieved a mean reduction in CPITN scores of 0.99 (95% CI: −1.225 to −0.162). In contrast, Kumar et al. [32] reported a mean decrease of 0.72, while Subedi et al. [87] observed improvements of 57.67% in plaque scores and 49.90% in the gingival index among 12–15-year-old schoolchildren following an oral health education intervention. Additionally, our intervention group experienced a mean reduction of −2.01 (95% CI: −2.355 to −1.656) in the SOHI, whereas Subedi’s trial achieved comparable reductions expressed in terms of percentage improvements. The differences between our study and those by Kumar et al. [32] and Subedi et al. [87] may stem from the fact that these studies employed a single mode of interactive oral education. In contrast, our study utilised a multi-modal, interactive approach combined with hands-on reinforcement over two months. This comprehensive approach likely contributed to the students’ adherence to oral hygiene behaviours.

Furthermore, the intervention group demonstrated a statistically significant reduction in the mean CPITN scores for all lower sextants (Right, Middle, Left), which indicated changes in brushing techniques or frequency by the participant students. The upper middle sextant, which usually encompasses the upper anterior teeth, is relatively more visible and easier to clean, yet it has also shown improvement in periodontal status.

On the other hand, regular breakfast consumption offers numerous health benefits, including improved cognitive performance, adequate nutrient intake, and positive effects on oral health [88,89]. Breakfast habits can influence the practice of oral hygiene [90]. Adolescents who consistently take their breakfast are more likely to develop structured daily routines, such as brushing their teeth after meals, suggesting that regular breakfast consumption may encourage healthier oral hygiene practices (ibid.). In this study, the analysis of dietary habits between the control and intervention groups revealed a statistically significant difference in breakfast consumption after the intervention, with the intervention group reporting higher breakfast intake. This finding aligns with health behaviour theories, such as the Health Belief Model, which dictates that individuals are more likely to adopt healthier behaviours when they perceive and understand their benefits. In this case, increased awareness about the systemic and oral health benefits of regular meals—reinforced through the educational sessions—may have contributed to improved breakfast habits. These results are consistent with Bahammam’s study findings [91], which reported that school health education programs significantly enhanced oral health behaviours and positively impacted broader health habits, including meal regularity.

The reduction in the number of cigarettes smoked per day is particularly noteworthy. Participants in the intervention group reported smoking about three fewer cigarettes daily than those in the control group. This decrease suggests behavioural change and may offer long-term health benefits, including a reduced risk of PD and other chronic illnesses. These findings align with the results of Prokhorov et al. in the U.S. Aspire project [92], which demonstrated that exposing young students from diverse backgrounds to multimodal interactive education led to significantly lower cigarette consumption. The study highlighted the effectiveness of incorporating interactive scenarios into educational curricula to influence youth smoking habits. Additionally, the frequency of water pipe smoking was notably lower in the intervention group. This result is consistent with studies by Nakkash et al. in Lebanon [93], Mays et al. in the United States [94], and Shahabi et al. in Iran [95]. Given the rising popularity of water pipe smoking among youth and the misconception that it is less harmful than cigarette smoking, this finding is significant. The educational program appears to have effectively addressed these misconceptions, contributing to a reduction in usage.

The analysis of net effects in this study demonstrated that the interactive oral health education program led to significant improvements in both clinical indices and health-related behaviours. Notably, the educational impact of this study program exceeded that observed in the study by Parihar et al. [96], despite our research being conducted over a shorter duration. This difference may be attributed to the multi-modal interactive approach and the incorporation of behavioural change components in education. In contrast, Parihar et al. utilised a more traditional method of health instruction, which did result in improvements, but to a lesser extent. In comparison, the findings of the present study are consistent with those of Shirahmadi et al. [97], who conducted a theory-based oral health intervention in Iran. Their results indicated significant behavioural improvements, with a 48.5% increase in twice-daily tooth brushing and a 64.2% increase in flossing. However, the current study demonstrated even more substantial clinical improvements, including a net reduction of 1.85 in SOHI and a 1.14 reduction in CPITN scores. This suggests that multimodal interactive education may yield similar results to theory-based education.

Despite the study’s strengths, some limitations should be considered, including the short follow-up period and potential bias in self-reported behaviours, such as smoking behaviours. While the program led to significant improvements, it remains uncertain whether these behaviours can be sustained in the long term. Follow-up evaluations at 6, 12, and 24 months would be valuable for assessing long-term effects. These evaluations will allow oral health policymakers to identify any relapse patterns and determine optimal timing for booster interventions.

## 5. Conclusions

Interactive school-based oral health education effectively improved students’ gingival status, oral hygiene status, and practices. Additionally, education had a positive impact on the participants’ students’ breakfast consumption, reduced intake of non-nutritious foods, and a decrease in both the frequency of smoking cigarettes and the use of water pipes. Notably, students who participated in this interactive education in Nablus city schools reported a significant reduction in the number of cigarettes consumed.

The transition from traditional oral health lectures to a multimodal, interactive approach—featuring videos, posters, live demonstrations, and hands-on group activities—significantly enhanced student engagement. This dynamic method fostered peer learning and motivation for better oral care. Ultimately, this shift played a central role in improving students’ oral health behaviours and periodontal outcomes.

## 6. Recommendations

It is essential to integrate an interactive oral health education model into school curricula. Policymakers should review national and regional oral health education standards, such as those for biology and personal development. They should then align the learning goals of the interactive sessions, such as “demonstrating proper tooth brushing techniques” and “describing the dangers of tobacco use”, with the required standards to ensure that all necessary skills are covered.

All materials should be compiled into an “Oral Health Toolkit.” This toolkit should include a facilitator’s guide, slide decks, videos, tooth models, puzzles, and worksheets, along with a simple checklist for each session that outlines the necessary materials and hands-on activities. Additionally, it is essential to organise a workshop for educators who will deliver or facilitate the instructional sessions.

Finally, identify six time slots of 45 to 60 min within the term, preferably during science classes, and conduct sessions regularly every two weeks to enhance learning and memory retention.

## Figures and Tables

**Table 1:** Geographical Distribution and Proportional Sample Allocation of Schools in Nablus.

Area/Nablus City	Total Schools	Proportion of Total	Sample Size (Proportional)
East	27	41.5%	6
Central	25	38.5%	6
West	13	20.0%	3
Total	65	100%	15

**Table 2:** Description of Oral Health Education Sessions: Objectives, Methods, and Evaluation Strategies.

No.	Session Title	Learning Objectives & Content	Teaching Methods	Instructional Tools	Evaluation Strategy
1	Anatomy & Physiology of the Oral Cavity and Clinical Presentation of PD.	Objectives: Identify the major anatomical structures of the oral cavity; Explain the normal function of periodontal tissues. Content: Overview of teeth, gingiva, periodontal ligament, alveolar bone; Signs and clinical presentation of PD.	Interactive lecture & Q&A	Smartboard, photographs, PowerPoint, dental models	Prepare a brief report on a family member’s oral condition
2	Prevention and Treatment of PD	Objectives: Describe primary preventive measures against PD; Compare non-surgical treatment options. Content: Oral hygiene techniques (brushing, flossing, mouthwash); Professional measures (scaling, polishing).	Group discussion & live demonstration under supervision	Whiteboard & markers, photographs, PowerPoint	Compile a list of barriers to effective oral hygiene
3	Maintenance and Follow-Up of Preventive Measures	Objectives: Outline a maintenance schedule for periodontal health; Apply problem-solving strategies to common barriers. Content: Recall intervals and re-evaluation Protocols.	Brainstorming & group discussion	Photos, preventive devices (toothbrush, floss, mouthwash), posters, puzzles	Develop a list of solutions to overcome identified barriers.
4	Daily Preventive Practices	Objectives: Practice correct brushing and interproximal cleaning techniques; Reinforce daily oral-care routines. Content: Live demonstrations of toothbrush and floss use; Hands-on practice with models and peer feedback.	Live demonstration under supervision & mini-lecture	Whiteboard, PowerPoint, tooth models, preventive devices (brush, floss, mouthwash)	Create a resource list for daily oral health information
5	Smoking and Its Harmful Effects on Periodontal Tissues	Objectives: Explain how tobacco smoking damages periodontal tissues; List short- and long-term oral health effects of smoking. Content: Chemical effects of nicotine and tar: Epidemiology of PD in smokers vs. non-smokers.	Live demonstration under supervision & lecture	Colourful PowerPoint slides with photos	Compile a resource list on the oral health impacts of smoking
6	Nutrition and Its Impact on Supportive Periodontal Tissues	Objectives: Analyse how different foods affect periodontal health; Recommend dietary adjustments to support periodontal tissues. Content: Role of sugars, acids, and antioxidants; Nutritional guidelines and examples of tooth-friendly diets.	Group discussion & live demonstration	Colourful PowerPoint slides with photos	Prepare a list of dietary guidelines and resources on nutrition and oral health.

**Table 3:** Socio-demographic Characteristics of the Study Sample at Baseline Phase.

Variable	Class	Control Group n (%)	Intervention Group n (%)	Chi-Square	*p*-Value
Sex	Male	244 (53.3)	233 (47.7)	2.889	0.089
	Female	214 (46.7)	255 (52.3)		
Type of School	Governmental	340 (74.2)	378 (77.5)	1.342	0.247
	Private	118 (25.8)	110 (22.5)		
Socioeconomic Status	Excellent/Very Good	36 (7.9)	54 (11.1)	5.472	0.140
	Good	95 (20.7)	79 (16.2)		
	Moderate	248 (54.1)	264 (54.1)		
	Low	79 (17.2)	91 (18.6)		
Number of Siblings	Only participant	46 (10.1)	40 (8.2)	6.141	0.293
	One	37 (8.1)	44 (9.0)		
	Two	104 (22.8)	88 (18.0)		
	Three	92 (20.1)	108 (22.1)		
	Four	145 (31.7)	178 (36.5)		
	Five	33 (7.2)	30 (6.1)		
Father’s Employment Status	Yes	360 (84.5)	403 (82.6)	3.712	0.156
	No	30 (6.6)	48 (9.8)		
	Don’t know	41 (9.0)	37 (7.6)		
Mother’s Employment Status	Yes	207 (45.2)	199 (40.8)	3.483	0.175
	No	217 (47.4)	260 (53.3)		
	Don’t know	34 (7.4)	29 (5.9)		
Father’s Education Level	No University	159 (34.7)	169 (34.6)	1.001	0.978
	University or Higher	299 (65.3)	319 (65.4)		
Mother’s Education Level	No University	160 (34.9)	229 (46.9)	14.033	* 0.000
	University or Higher	298 (65.1)	259 (53.1)		

* *p* < 0.05 is considered statistically significant.

**Table 4:** The Comparison of Overall Mean of CPITN and S-OHI Scores Between Control and Intervention Groups at Baseline and after 2 Months of Education.

Variable	Group	Control Mean ± SD	Intervention Mean ± SD	MD	t-Value	*p*-Value	CI (95%) (Lower–Upper)	
CPI	Baseline	10.85 ± 2.85	10.99 ± 2.766	0.139	0.829	0.407	0.190	0.476
	After 2 months	11.00 ± 2.582	10.00 ± 2.639	−1.000	5.700	0.000	1.314	−0.641
		P = 0.242	P = 0.000					
		T = 1.173	T = 5.595					
		CI (−0.142–0.561)	CI (−0.162–1.225)					
SOHI	Baseline	12.69 ± 2.619	12.90 ± 3.103	0.208	1.091	0.276	−0.166	−0.582
	After 2 months	12.53 ± 2.466	10.89 ± 2.779	−1.637	−9.424	0.000	−1.978	−1.296
		P = 0.365	P = 0.000					
		T = 0.906	T = −11.279					
		CI (−0.610–0.188)	CI (−2.355–−1.656)					

Note. MD = Mean Differences.

**Table 5:** Comparison of Mean of CPITN Scores Across Mouth Sextants Between Control and Intervention Groups After 2 Months of Education.

Sextant	Group	Mean ± SD	MD	t-Value	*p*-Value	95% C. I.	
						Lower	Upper
Upper right	control group	2.02 ± 0.699					
	intervention group	2.00 ± 0.805	−0.018	−0.353	0.724	−0.116	0.080
Lower right	control group	1.54 ± 0.617	−0.215	−5.384	0.000	−0.293	−0.137
	intervention group	1.33 ± 0.602					
Upper middle	control group	1.94 ± 0.594	−0.323	−7.621	0.000	−0.406	−0.240
	intervention group	1.61 ± 0.682					
Lower middle	control group	2.19 ± 0.756	−0.176	−3.395	0.001	−0.278	−0.074
	intervention group	2.02 ± 0.814					
Upper left	control group	1.86 ± 0.705	−0.025	−0.522	0.602	−0.120	0.069
	intervention group	1.84 ± 0.756					
Lower left	control group	1.50 ± 0.573	−0.221	−5.892	0.000	−0.295	−0.148
	intervention group	1.28 ± 0.572					

Note. MD = Mean Differences.

**Table 6:** Comparison of the Mean of S-OHI Scores Across Mouth Sextants Between Control and Intervention Groups After 2 Months of Education.

Sextant	Group	Mean ± SD	MD	t-Value	*p*-Value	95% C. I.	
						Lower	Upper
Upper right	control group	2.27 ± 0.651	−0.217	−4.532	0.000	−0.311	−0.123
	intervention group	2.06 ± 0.786					
Lower right	control group	1.79 ± 0.639	−0.336	−7.964	0.000	−0.418	−0.253
	intervention group	1.45 ± 0.645					
Upper middle	control group	2.06 ± 0.552	−0.284	−6.541	0.000	−0.370	−0.199
	intervention group	1.77 ± 0.736					
Lower middle	control group	2.40 ± 0.751	−0.379	−7.506	0.000	−0.478	−0.280
	intervention group	2.03 ± 0.783					
Upper left	control group	2.25 ± 0.676	−0.154	−3.324	0.001	−0.245	−0.063
	intervention group	2.10 ± 0.730					
Lower left	control group	1.75 ± 0.555	−0.267	−6.620	0.000	−0.346	−0.188
	intervention group	1.49 ± 0.656					

Note. MD = Mean Differences.

**Table 7:** Comparison of Overall Oral Hygiene Practices, Diet Habits, and Smoking Behaviours between Control and Intervention Groups at Baseline and After 2 Months of Education.

Variable	Group	Control Mean ± SD	Intervention Mean ± SD	t-Value	*p*-Value
Overall oral hygiene practices	Baseline	14.53 ± 5.426	13.60 ± 6.548	1.968	0.066
	After 2 months	14.69 ± 5.396	17.86 ± 5.931	8.461	0.000
		P = 0.047	P = 0.001		
		T = 1.988	T = 11.921		
Overall Diet habits	Baseline	35.36 ± 3.780	35.08 ± 5.2268	0.603	0.563
	After 2 months	35.85 ± 3.655	37.20 ± 4.661	4.839	0.000
		P = 0.012	P = 0.001		
		T = 2.524	T = 6.304		
Overall smoking behaviours	Baseline	6.11 ± 3.429	6.94 ± 3.441	−3.459	0.071
	After 2 months	6.40 ± 3.606	7.67 ± 3.573	5.400	0.000
		P = 0.356	P = 0.005		
		T = 0.924	T = 2.813		
The Sum of Overall Practices	Baseline	55.26 ± 8.114	54.75 ± 10.426	0.645	0.645
	After 2 months	56.94 ± 8.168	62.73 ± 9.834	9.644	9.644
		P = 0.000	P = 0.000		
		T = 3.741	T = 17.436		

**Table 8:** Comparison of Oral Hygiene and Dental Visit Practices between Control and Intervention Groups.

Variable	Control Mean ± SD	Intervention Mean ± SD	t	*p*-Value
Brushing status	0.83 ± 0.375	0.93 ± 0.254	4.851	0.000
Frequency of teeth brushing	0.98 ± 0.593	1.21 ± 0.563	6.140	0.000
Duration of brushing in minutes	0.70 ± 0.609	1.14 ± 0.859	8.760	0.000
Use a modified Bass-brushing technique	1.094 ± 0.29242	1.9431 ± 0.4986	−16.396	0.000
Using a medium-sized bristle toothbrush	1.1471 ± 0.354	1.8212 ± 0.485	−16.377	0.000
Dentist visiting	1.10 ± 0.294	1.15 ± 0.355	−2.477	0.013
Regular visits to the dentist	1.077 ± 0.268	1.13 ± 0.344	−2.812	0.005

**Table 9:** Comparison of Dietary Habits Between Control and Intervention Groups.

Variable	Control Mean ± SD	Intervention Mean ± SD	t	*p*-Value
Breakfast Consumption Habits	5.825 ± 2.346	6.2500 ± 2.442	−2.703	0.007
Frequency of eating nutritious food	21.000 ± 2.290	21.278 ± 3.268	−1.537	0.125
Frequency of eating non-nutritious food	9.046 ± 0.925	9.067 ± 1.171	−0.254	0.800

**Table 10:** Comparison of Smoking Habits Between Control and Intervention Groups After 2 Months of Education.

Variable	Control Mean ± SD	Intervention Mean ± SD	t	*p*-Value
Cigarette Smoking Status	1.56 ± 0.501	1.50 ± 0.500	2.043	0.040
Smoking Frequency	2.663 ± 0.638	2.6213 ± 0.713	0.656	0.515
The number of cigarettes	11.05 ± 4.218	8.04 ± 3.560	7.800	0.000
Water pipe smoking status	1.14 ± 0.349	1.13 ± 0.331	0.740	0.001
Water pipe smoking frequency	2.673 ± 0.890	2.201 ± 0.966	7.778	0.000

**Table 11:** The net Effect of education on Periodontal and oral hygiene Status and Related Behaviours.

Index/Domain	Intervention Pre (Mean)	Intervention Post (Mean)	Intervention Change	Control Pre (Mean)	Control Post (Mean)	Control Change	Net Effect
CPITN	10.99	10.00	−0.99	10.85	11.00	0.15	−1.14
S-OHI	12.90	10.89	−2.01	12.69	12.53	−0.16	−1.85
Oral Hygiene practices	13.60	17.86	4.26	14.53	14.69	0.16	4.10
Diet Habits	35.08	37.20	2.12	35.36	35.85	0.49	1.63
Smoking Behaviors	6.94	7.67	0.73	6.11	6.40	0.29	0.44
Sum of All Practices	54.75	62.73	7.98	55.26	56.94	1.68	6.30

## Data Availability

The data presented in this study are available on reasonable request from the corresponding author. The data are not publicly available due to privacy and ethical restrictions.

## Supplementary Materials

The following supporting information can be downloaded at: https://www.mdpi.com/article/10.3390/children12101302/s1, Figure S1.

## Abbreviations

The following abbreviations are used in this manuscript:

Abbreviation	Full Term
PD	Periodontal Disease
CPITN	Community Periodontal Index for Treatment Needs
S-OHI	Simplified Oral Hygiene Index
OHI-S	Oral Hygiene Index–Simplified
SPSS	Statistical Package for the Social Sciences
GI	Gingival Index
PI	Plaque Index
WHO	World Health Organisation
ICC	Intraclass Correlation Coefficient
Epi Info	Epidemiological Information Software
CI	Confidence Interval
SD	Standard Deviation
MD	Mean Difference

## Appendix A

Coding system

Section 2: Oral Hygiene Practices			
The question	Responses	Old codes	New scores
B.1—Do you brush your teeth?	Yes	1	1
	No.	2	0
B1.1—How often do you usually brush your teeth?	Once a day	1	1
	Twice a day or more	2	2
	Sometimes	3	3
B.1.3—How long do you spend brushing your teeth?	A minute or less	1	1
	Two minutes	2	2
	More than two minutes	3	3
B.1.4—How do you brush your teeth?	Bass method	1	2
	Scrubbing method	2	1
	Modified Bass method	3	3
	Other	4	0
B.1.5—What kind of bristles does your toothbrush have?	Soft bristles	1	2
	Medium bristles	2	3
	Hard bristles	3	1
	I am not sure	4	0
B.1.6—How often do you change your toothbrush?	Every month	1	3
	Every two months	2	2
	Every three months	3	1
	Other	4	0
B.1.7—Do you use other oral and dental cleaning tools besides the toothbrush?	Dental floss	1	3
	Interdental brush	2	2
	Mouthwash	3	1
	Do not use	4	0
B.1.8—Do you visit the dentist in your life?	Yes.	1	1
	No.	2	0
B.1.9—How did you visit the dentist?	Regularly	1	3
	Sometime	2	2
	Pain	3	1
B.1.10—When was the last time you visited the dentist?	Before 6 months	1	3
	Before 6–12 months	2	2
	Before 1–2 years	3	1
	Before 3–5 years	4	0
B.1.11—If you visited the dentist, what was the purpose of your visit?	Pain	1	0
	Recommendation from a friend or relative	2	1
	Recommendation from another dentist	3	2
	Routine dental check-up	4	3
	Teeth and gum cleaning	5	4
Section 3: Diet Habits			
C.1.1—Do you eat breakfast on weekdays?	Never	1	0
	Once a week	2	1
	Twice a week	3	2
	Three times a week	4	3
	Four times a week	5	4
	Every day	6	5
C.2.1—Do you have breakfast on weekends?	Never	1	0
	Once a week	2	1
	Every weekend	3	2

C.2—How often do you eat or drink the following items? (Tick one box for each item by marking an “X” under the option that applies to you in each row):

**Item**	**Never**	**Less Than Once a Week**	**Once a Week**	**2–4 Times a Week**	**6 Times a Week**	**Once a Day**	**More Than Once a Day**
Fruits	0	1	2	3	4	5	6
New code	0	1	2	3	4	5	6
Vegetables	0	1	2	3	4	5	6
New code	0	1	2	3	4	5	6
Sweets (e.g., candy or chocolate)	0	1	2	3	4	5	6
New code	6	5	4	3	2	1	0
Soft drinks or any sugar-containing beverages	0	1	2	3	4	5	6
New code	6	5	4	3	2	1	0
Milk and dairy products	0	1	2	3	4	5	6
New code	0	1	2	3	4	5	6
Red meat	0	1	2	3	4	5	6
New code	0	1	2	3	4	5	6
Fish	0	1	2	3	4	5	6
New code	0	1	2	3	4	5	6
Water	0	1	2	3	4	5	6
New code	0	1	2	3	4	5	6
Nuts	0	1	2	3	4	5	6
New code	0	1	2	3	4	5	6
Pastries	0	1	2	3	4	5	6
New code	6	5	4	3	2	1	0
Section 4: Smoking behaviours							
The question		Responses			Old codes		New scores
D.1—Do you currently smoke (at least one cigarette)?		Yes			1		2
		No			2		0
D.2—How frequently do you smoke cigarettes or use other tobacco products?		Every day			1		1
		At least once a week but not every day			2		2
		Less than once a week			3		3
		I don’t smoke			4		4
D.2.2—If you smoke cigarettes, how many cigarettes do you usually smoke per week?		26 and over					0
		21–25 cigarettes					1
		16–20 cigarettes					2
		11–15 cigarettes					3
		6–10 cigarettes					4
		0–5 cigarettes					5
D.3—Have you ever smoked a waterpipe (hookah)?		Yes			1		2
		No			2		0
D.4—How often do you currently smoke a water pipe?		Every day			1		1
		At least once a week but not every day			2		2
		Less than once a week			3		3
		I don’t smoke waterpipe			4		4
