# Supplementary figures and images for: Effectiveness of an Interactive School-Based Oral Health Educational Program on Periodontal Status Among Palestinian Adolescents: An Intervention Study
Source: Children (Basel). 2025 Sep 26;12(10):1302. doi: 10.3390/children12101302 (PMC12563124; doi:10.3390/children12101302)

# Schematic of the School-Based Oral-Health Education Intervention

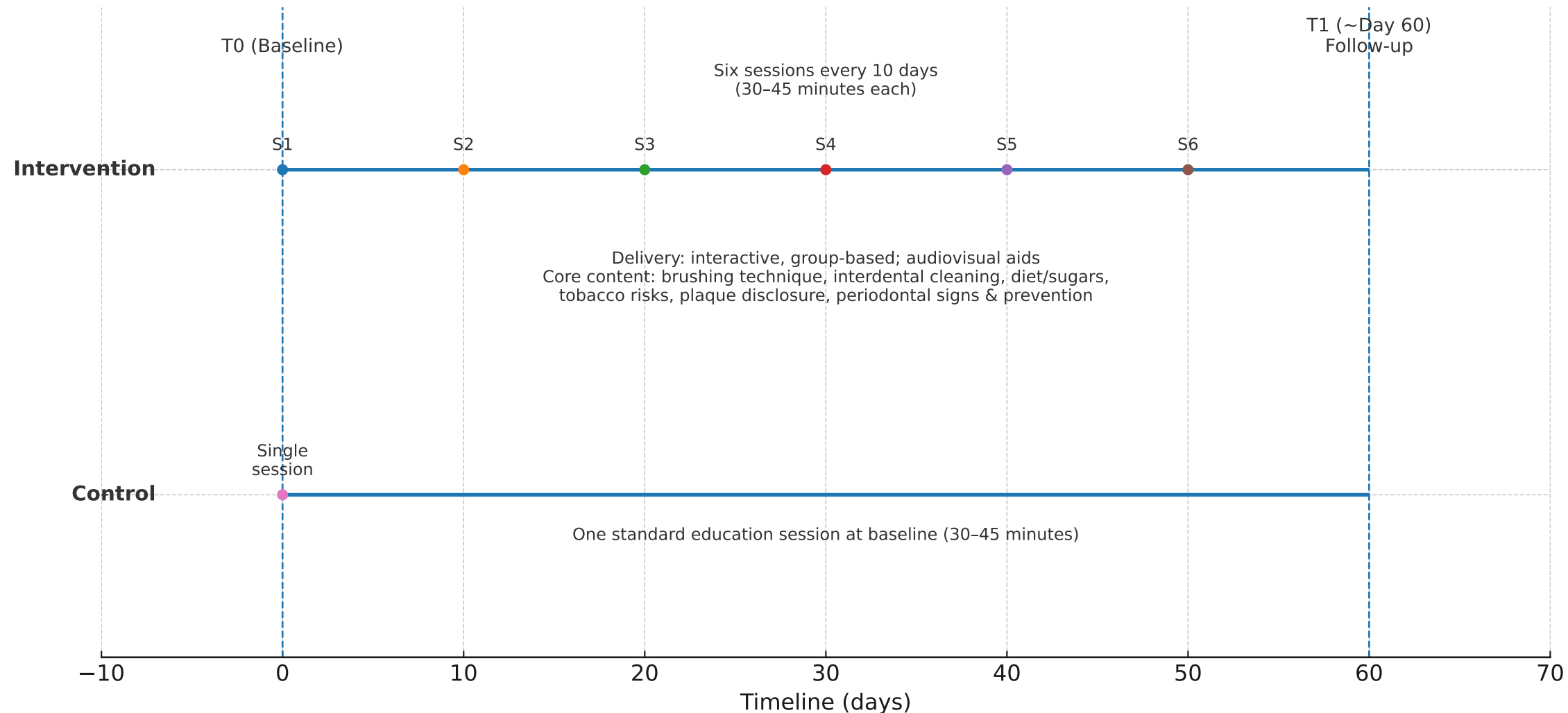

Supplement: Supplementary file 1 [file children-12-01302-s001.zip › children-3815443-supplementary.pdf]
